# EHMTI-0310. Headache in children with Sturge-Weber syndrome

**DOI:** 10.1186/1129-2377-15-S1-C52

**Published:** 2014-09-18

**Authors:** P Prabhakar, L Arkush, R Scott, S Aylett

**Affiliations:** 1 Neurology, Great Ormond Street Hospital, London, UK

## Introduction

Patients with Sturge-Weber syndrome can have varying degrees of neurological impairment – Epilepsy, hemiparesis, visual field defects, cognitive deficits, endocrine, psychiatric and ophthalmological involvement. Headaches affect 30-45% of patients with SWS’ (Thomas-Sohl et al., 2004). Over half patients feel that headaches have equal or greater impact on life than seizures.

## Aims

To describe the frequency, associations and disability of headaches in children with Sturge Weber Syndrome from a tertiary specialist clinic.

## Methods

A computer generated list of all patients seen at Great Ormond Street Hospital Sturge-Weber Clinic over a 10 year period was compiled. Clinical and Demographic information including epilepsy diagnosis, developmental delay, glaucoma, focal weakness, brain involvement, drugs prescribed, headache history were collected.

## Results

88 patients’ notes reviewed. 4 excluded due to incomplete information. n= 84. 31 patients (37%) have documented reports of ≥1 headache and screaming episodes. Mean age of onset = 7 years, 3 months (SD 4 years, 1 month; range 5 months – 15 years). Duration ranged from 3 minute-long episodes to 10 days. Precipitating factors: head trauma, Seizures, behavioural problems, viral infections, Pilocarpine, noisy environments and school lessons.

## Conclusion

35% headache patients have headaches more than once a month. 25% of patients describe headaches impacting on their activities. It is unclear whether they fit migraine headache criteria. Female sex, children with glaucoma, hemiplegia and recent seizures tend to be over represented in the headache group. The significance of this needs further analysis by a prospective study.

No conflict of interest.

